# Formulation and Evaluation of Gelatin Micropellets of Aceclofenac: Effect of Process Variables on Encapsulation Efficiency, Particle Size and Drug Release

**DOI:** 10.4103/0250-474X.49126

**Published:** 2008

**Authors:** S. K. Sahoo, M. K. Jena, S. Dhala, B. B. Barik

**Affiliations:** University Department of Pharmaceutical Sciences, Utkal University, Vani-Vihar, Bhubaneswar-751 004, India

**Keywords:** Aceclofenac, micropellets, gelatin, cross linking, process variables

## Abstract

In the present study aceclofenac-gelatin micropellets were prepared by the cross linking technique using gluteraldehyde as cross linking agent and characterized by X-ray diffractometry, differential scanning calorimetry and scanning electron microscopy. The effect of drug: polymer ratio, temperature of oil phase, amount of gluteraldehyde and stirring time was studied with respect to entrapment efficiency, micropellet size and drug release characteristics. Spherical micropellets having an entrapment efficiency of 57% to 97% were obtained. Differential scanning calorimetric analysis confirmed the absence of any drug-polymer interaction. The micromeritic studies of micropellets show improved flow property. The entrapment efficiency, micropellet size and drug release profile was altered significantly by changing various processing parameters.

Natural polymer such as gelatin has been extensively used for the preparation of particulate drug delivery systems by virtue of its biocompatibility and biodegradability along with a total absence of toxicity or allergic problems^1^. Being a water soluble polymer, gelatin has to be chemically cross linked to become insoluble at 37°. Aldehyde derivatives such as formaldehyde; glutaraldehyde or other bifunctional reactants have been used to produce insoluble gelatin microspheres^2^,^3^. In the present study a non-steroidal antiinflammatory drug aceclofenac has been chosen as a model drug^4^ (half-life of 4 h) and the effect of various processing parameters such as drug: polymer ratio, temperature of oil phase, amount of gluteraldehyde and stirring time on various physical properties and *in vitro* drug release were studied. Micropellets were also evaluated for drug polymer interactions by DSC, crystallinity by X-RD and surface structure by scanning electron microscopy (SEM).

The aceclofenac micropellets were prepared by cross linking technique using gelatin polymer. The gelatin (1.0 g) was soaked in 12 ml of distilled water for 15 min. It was then heated on a water bath to 60-65° until it completely dissolved. The drug (1.0 g, previously passed through no. 80 sieve) was added to the gelatin solution with stirring. The drug loaded micropellets were formed by dropping the drug polymer dispersion by a jacketed glass syringe (20 G, provided with hot water supply at 60-65°) in to previously cooled 300 ml sunflower oil (10°, maintained by ice salt freezing mixture) containing 0.5 ml of Span 20 as dispersing agent, with constant stirring at 200 rpm. The gluteraldehyde (25% v/v) solution (1.5 ml) was added after 1 h to crosslink the gelatin, and the stirring was further continued for 2.5 h. The resulting micropellets were decanted, freed of sunflower oil by repeated washings with ice cold isopropyl alcohol (150 ml) and finally air dried over a period of 24 h. Ten batches of aceclofenac micropellets were prepared in triplicate (Table 1) by altering one of the four process variable at a time, keeping the others constant as mentioned above.

**TABLE 1:** EFFECT OF PROCESSING PARAMETERS ON DRUG LOADED MICROPELLETS

Processing and formulation parameters	Batch code	% Yield*	Mean particle size* (μm)	EE* (%)	Carr's index*
Polymer-drug Ratio					
1:0.25	F_1_	92.80	498. 82	71.45	5.26
1:0.33	F_2_	80.94	530.17	89.55	4.34
1:0.5	F_3_	75.00	561.55	91.46	4.76
1:1	F_4_	75.10	563.43	97.00	4.00
Amount of gluteraldehyde (ml)					
3.5	F_5_	80.55	474.08	88.09	4.76
2.5	F_6_	71.75	467.23	87.28	5.00
0.5	F_7_	81.70	482.63	64.60	5.00
Stirring time (h)					
6	F_8_	69.75	531.83	92.94	5.00
5	F_9_	68.90	520.63	94.96	10.00
Oil phase temperature*					
60	F_10_	92.80	498.83	57.30	11.11

* Effect of processing parameters on % yield, mean particle size, entrapment efficiency and carr's index of drug-loaded micropellets. Each observation is the mean of three determinations. EE stands for entrapment efficiency.

The flow properties of the prepared micropellets were determined by measuring the Carr's index^5^. Micropellets were separated into different size fractions by sieving for 10 min using mechanical sieve shaker (Cuprit Electrical Co. India) containing standard sieves having apertures of 1000, 710, 500, 355, 250 and 180 μ, respectively. The particle size distribution of the micropellets for all the formulations was determined and mean particle size of micropellets was calculated. The % drug content in micropellets were determined in phosphate buffer, pH 7.5 at 274 nm using Systronic 2101 UV/Vis spectphotometer. The DSC analysis of pure drug, blank micropellets, and drug loaded micropellets was carried out using PerkinElmer DSC model (PerkinElmer Inc, Boston, MA) to evaluate any possible drug polymer interaction. Powder X-RD patterns were recorded on Rigaku, Japan (Model-MenifleX) using Ni-filtered, Cuk α radiation, a voltage of 30 kv and a current of 25 ma. The scanning rate employed was 2° min^−1^, over the 4° to 40° diffraction angle (2θ) range. Jeol JSM–5200, scanning electron microscope was used to characterize surface topography of prepared micropellets. The *in vitro* dissolution studies were carried out in 500 ml of phosphate buffer, pH 7.5, maintained at 37±0.5° and 100 rpm by using United States Pharmacopoeia basket type dissolution test apparatus (Lab India, Disso-2000, Mumbai, India) under sink conditions^6^–^8^.

The resulting micropellets obtained by above procedure were free flowing in nature as Carr's index of all formulation lies in the range 4-12 (Table 1). Entrapment efficiency was found to increase by increasing polymer:drug ratio from 1:0.25 to 1:1 (Table 1). The same case was noticed with mean particle size. At higher polymer-drug ratio, large droplets were formed due to increased viscosity of polymeric phase and, hence an increase in the mean particle size. The temperature of the oil phase is one of the important process parameters, as good entrapment efficiency was found at low temperature (10°), which also contributed to hardness of micropellets. A sharp endothermic (T_peak_ =153.67°) was observed for aceclofenac at the room temperature corresponding to its melting point 150-158° (fig. 1). In case of gelatin, exothermic peak was observed in the temperature range of 200-250°. The characteristic, well-recognizable thermal profile of the drug appeared at the temperature corresponding to its melting point in the aceclofenac gelatin micropellets showing thermal peak at 151.90° but with the loss of its sharp appearance. It appears that there is a significant reduction of drug crystallinity in the polymer matrix. The X-ray powder diffraction patterns of pure drug and formulation containing aceclofenac (fig. 2) reveals that the intensity of the peaks for the pure drug was sharp, but when it was incorporated into the polymer matrix, the drug peaks showed a loss of sharpness due to decreased crystallinity of the drug. SEM of drug-loaded micropellets revealed that the micropellets were spherical in shape. The drug release from micropellets prepared at lower drug: polymer ratios was faster than that of micropellets prepared at higher drug-polymer ratios because of the small size of the micropellets at lower drug: polymer ratios, which provided a large surface area for faster drug release (data not shown). Micropellets prepared at 60° showed faster drug release than the micropellets prepared at 10° as shown in (fig. 3). This result may be accredited to the deterioration of the micropellet wall by gluteraldehyde at higher temperature, which may lead to rapid release of drug with initial burst effect. It was found that increase in amount of cross linking agent decreases the release time. This result may be attributed to the higher degree of cross linking of gelatin at higher gluteraldehyde concentration, which retarded drug release from the pellets (fig. 3). The micropellets treated with gluteraldehyde for a longer period of time showed slower drug release as compared to micropellets treated for a shorter period of time as evident from (fig. 3). This may be due to improvement in hardness of micropellets at longer exposure time.

**Fig. 1:**
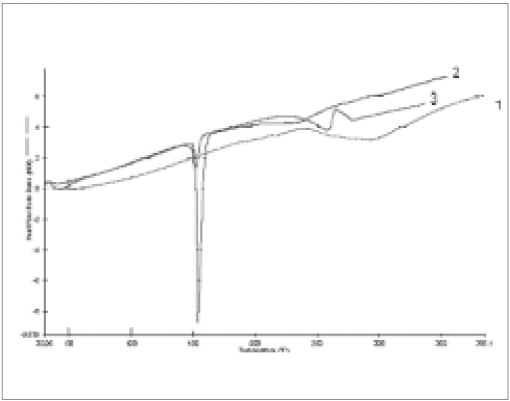
DSC thermograms of aceclofenac, aceclofenac-loaded micropellets and blank gelatin microspheres. (1) Blank gelatin micropellets, (2) aceclofenac loaded micropellets and (3) aceclofenac.

**Fig. 2:**
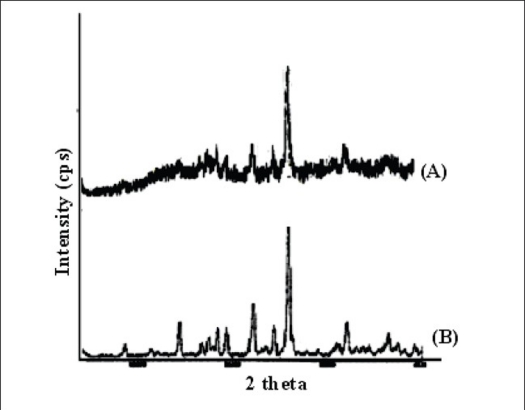
X-RD curves of aceclofenac loaded micropellets and aceclofenac. (A) Aceclofenac-loaded micropellets and (B) aceclofenac

**Fig. 3:**
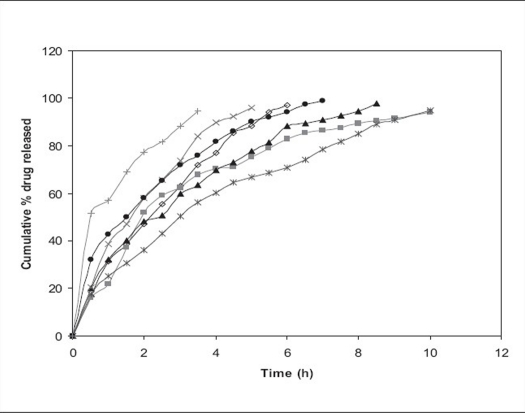
Release of aceclofenac from formulations Effect of amount of gluteraldehyde, stirring time and temperature of oil phase on the cumulative % drug release of aceclofenac from formulations (–◊–) F4, (–■–) F5, (–▲–) F6, (–X–) F7, (–*–) F8, (–●–) F9 and (–+–) F10. All the values are mean of three observations.
